# Pearls and pitfalls in lung cancer staging

**DOI:** 10.1259/bjro.20200019

**Published:** 2020-07-08

**Authors:** Lucian Beer, Ankush Jajodia, Helmut Prosch

**Affiliations:** 1 Department of Biomedical Imaging and Image-guided Therapy, Medical University of Vienna, Vienna, Austria; 2 Department of Radiology and Cancer Research UK, Cambridge, England, UK; 3 Rajiv Gandhi Cancer Institute and Research Centre, Delhi, India

## Abstract

Lung cancer is the third most common cancer in the UK and is the leading cause of death. Radiology plays a central role in the diagnostic work-up of patients with suspected and known lung cancer. Tumour assessment includes both local staging, as well as distant staging. Local staging objectives include the assessment of technical resectability with regard to the evaluation of tumour size and invasion of surrounding structures. Distant staging objectives aim to identify distant metastasis in lymphatic and extra lymphatic tissues. CT, positron emission tomography/CT, MRI, and ultrasound are routinely used imaging techniques for staging in patients with lung cancer. In this review, we will consider the pitfalls of these examinations that radiologists potentially face during the work-up of patients with lung cancer.

## Introduction

Radiologists are fundamentally involved in the diagnosis and treatment decision-making in patients with lung cancer and see patients with lung cancer at various stages of their journey. The radiologist is frequently the first contact, often at time of their initial diagnosis, which can either be during lung cancer screening examinations or during diagnostic examinations. Patients are then discussed within the multidisciplinary team (MDT) and in many cases, the next step in the diagnostic process is tumour tissue sampling, in which radiologists are also involved. Once the diagnosis of lung cancer is established, patients are staged based on the anatomical tumour extent. Finally, imaging examinations are used for treatment response assessment and for evaluation of disease recurrence. Each step comprises peculiarities and challenges for radiologists. In this review, we will focus on the pearls and pitfalls of lung cancer staging from a radiology perspective.

### Eighth edition of the TNM classification of malignant tumours

Since 01 Jan 2017, the eighth edition of the TNM staging system, as proposed by the International Association for the Study of Lung Cancer (IASLC), should be used to stage non-small cell lung cancer (NSCLC) and small cell lung cancer (SCLC). This staging system is based on a database analysis of 94,708 cases donated from 35 sources in 16 countries around the world between 1999 and 2010.^1^ Of these, 70,967 validated cases were used for final analysis. The eighth edition addresses several limitations of the seventh edition, although several issues persist.

In the TNM staging system, the T descriptor describes the local tumour extent, the N descriptor the involvement of hilar or mediastinal lymph nodes, and the M descriptor intra- and extrathoracic distant metastases.

### T-descriptors

The T descriptor indicates the size of the primary tumour and its extension into neighbouring structures, such as the chest wall or the mediastinum. Furthermore, the T descriptor is also used to describe ipsilateral pulmonary metastases.

Based on their size, tumours are categorized as T1 tumours with a maximum diameter of less than 3 cm, T2 tumours with a maximum diameter between 5 cm and 7 cm, and T3 tumours with a maximum diameter of more than 7 cm. T1 and T2 tumours are further subclassified in T1a, T1b, and T1c, and T2a and T2b in 1 cm increments to allow an even better prognostic classification.^2^

### How to measure tumour size

**Figure 1.**
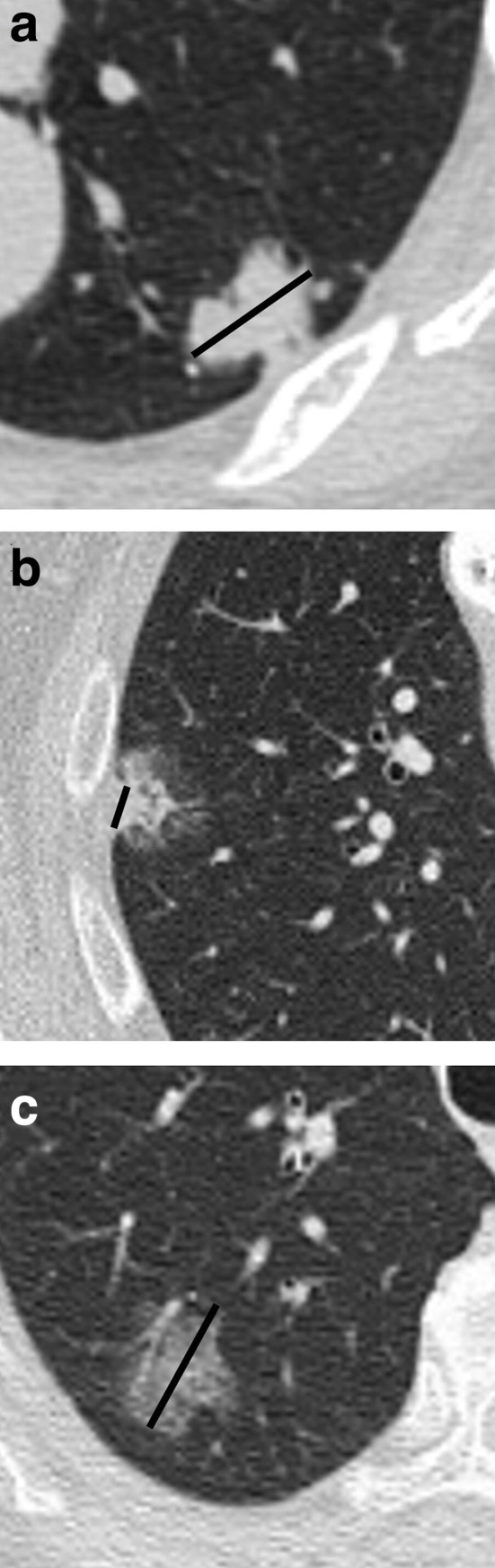
Axial non-enhanced CT showing (a) a solid nodule in the left lower lobe compatible with a biopsy-proven invasive adenocarcinoma. It is recommended that the solid part be measured (black line). The solid part represents the invasive component of the tumour and should be used to define the T classification for staging. In (b), a part-solid tumour in the right upper lobe is shown. It is recommended that the solid dimension (black line) of the tumour for the T-descriptor be used. In (c), a pure ground-glass nodule is shown. The largest dimension of the ground-glass tumour should be measured and should be classified as an adenocarcinoma *in situ* (cTis).

The TNM manifest defines the way in which radiologists should measure lung cancer for TNM staging. For solid tumours, the single largest dimension measured in one of the three standard planes (axial, coronal, sagittal) using thin sections (1 mm) should be measured and used for the T descriptor (Figure 1A).^3,4^ In part-solid tumours, the largest tumour dimension, including the ground-glass (GG) part, should be recorded, but the staging is based on the largest diameter of the solid component. The solid component in part-solid, non-mucinous, lung adenocarcinomas correlates with an invasive adenocarcinoma pattern, while the GG component correlates with a lepidic growth pattern.^3^

Pure GG lesions with the largest diameter between 0.5 cm and 3 cm are classified as T1_is_, indicating that such pure GG lesions are most likely *in situ* tumours (Figure 1C). Pure GG lesions smaller than 0.5 cm are not staged, as the probability of such lesions being malignant is very low. Pure GG lesions larger than 3 cm are classified as T1a.

**Figure 2.**
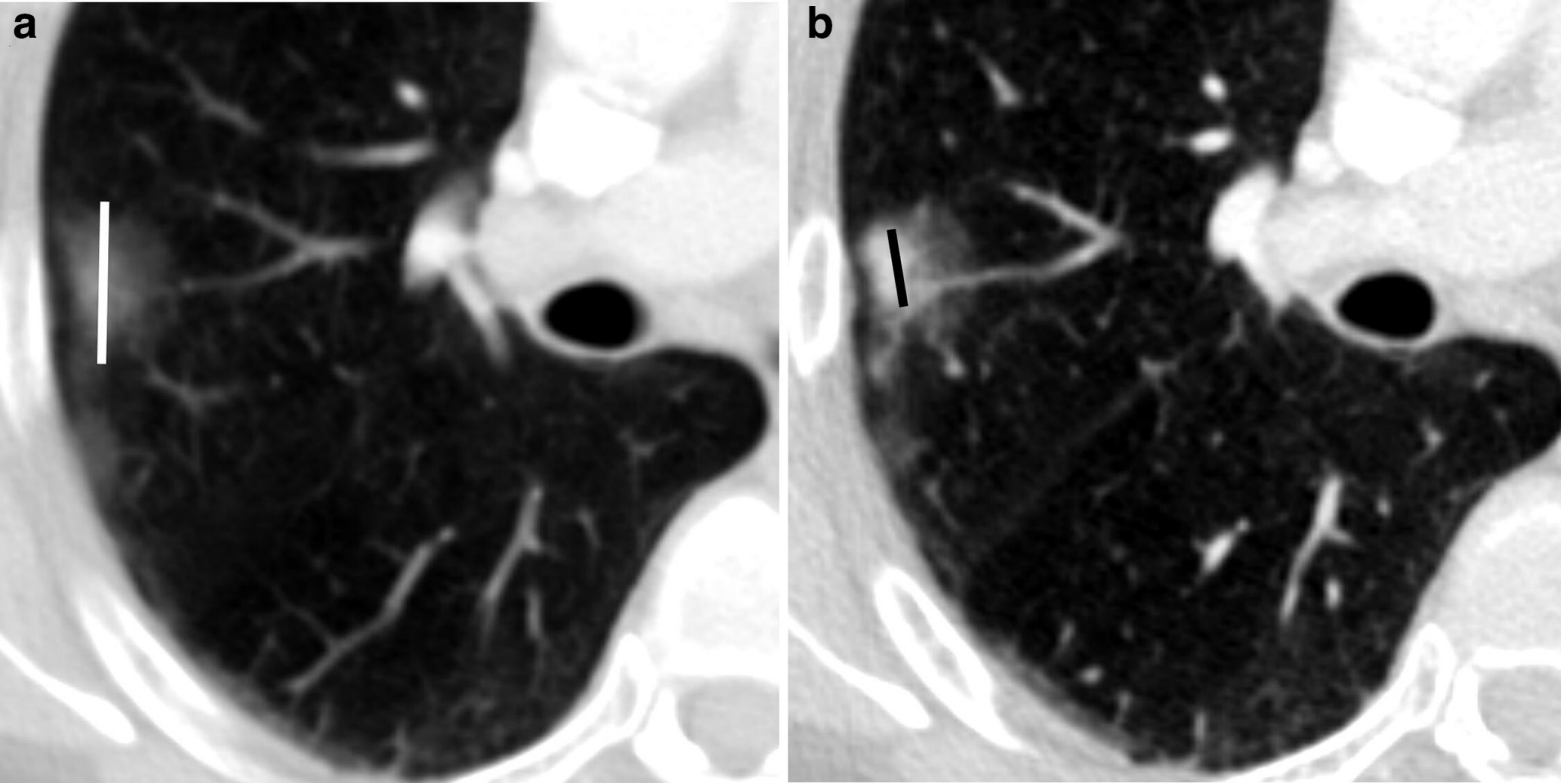
Axial contrast-enhanced CT shows an adenocarcinoma of the right upper lobe. Depending on the slice thickness, the tumour displays pure ground-glass properties on (a) 3 mm reconstructions or solid components on (b) 1 mm reconstructions. It is, therefore, recommended to use only 1 mm reconstructions with sharp reconstruction kernels for lung cancer staging.

Tumour size should be recorded in centimetres and include millimetre increments. It is recommended that thin slices (1 mm) be used, as thick-slice reconstructions (1.5 to 5 mm) could mask small solid components. These tumours would, therefore, mistakenly be classified as pure GG tumours (Figure 2).

In the rare cases of part-solid tumours with several solid components, the radiologist should measure the long axis of the largest solid component.^3^

### Atelectasis

In tumours in which the obstructive effect of the tumour leads to an atelectasis or a post-obstructive pneumonia, a reliable determination of tumour size is not possible. Consequently, partial or total atelectasis of a lobe or entire lung side or a post-obstructive pneumonia are defined as T2.

**Figure 3.**
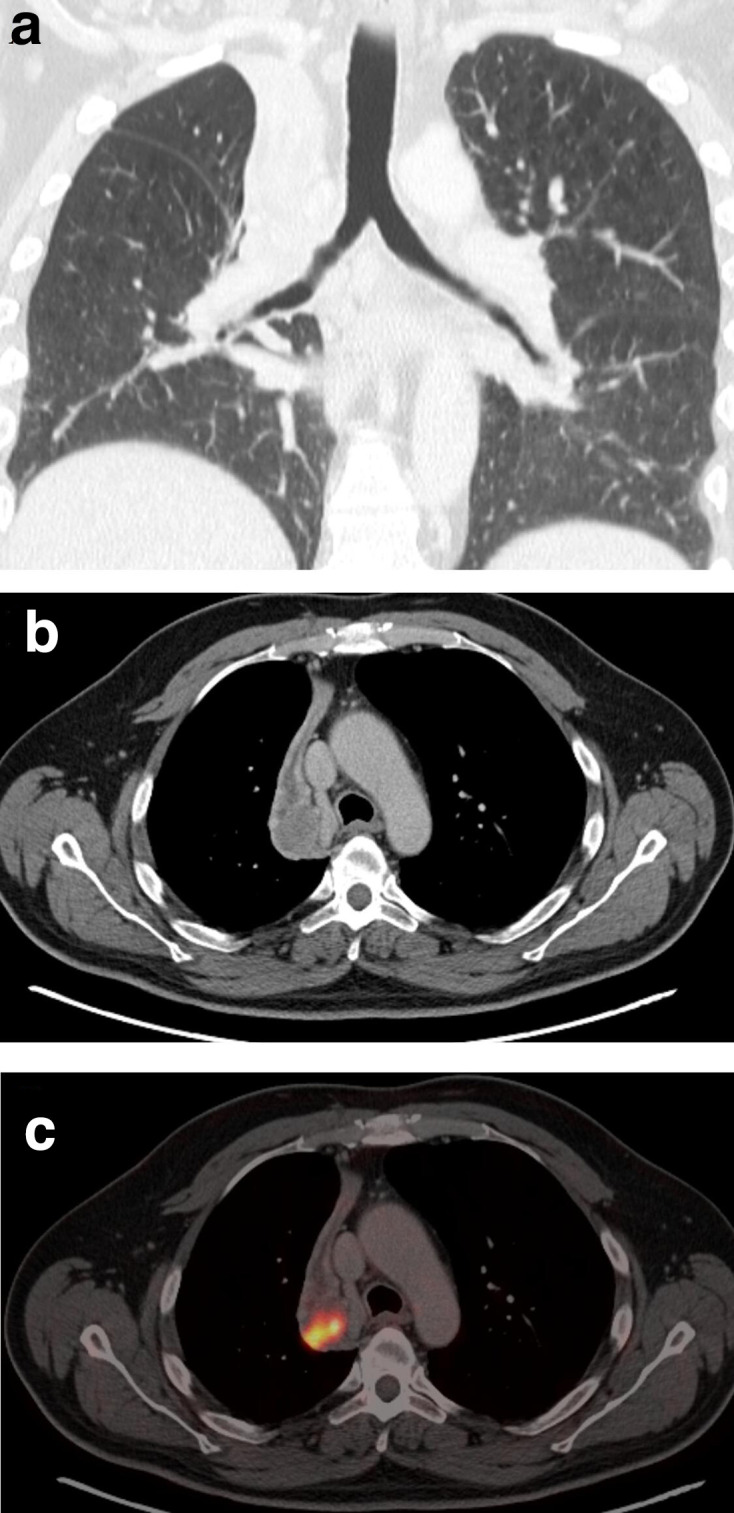
(a) A 65-year-old patient with NSCLC and right upper lobe atelectasis. On the contrast-enhanced CT (b) an inhomogeneous mass is seen at the level of the right hilum that obliterates the right upper lobe bronchus. The exact tumour diameter cannot be assessed accurately. (c) FDG -PET/CT shows focally increased FDG uptake in the tumour, while the post-stenotic atelectatic lung shows only a moderate FDG uptake. In this patient, the metabolic information helps to correctly assess tumour dimension. FDG, fludeoxyglucose; NSCLC,non-small cell lung cancer; PET, positron emission tomography

In patients with central tumours leading to an atelectasis, in which a delineation of tumours is necessary to limit the radiation field, PET/CT or PET/MRI are of help in identifying the obstructing tumour^5^ (Figure 3).

### Infiltration of the chest wall or the diaphragm

While an infiltration of the chest wall is defined as T3, an infiltration of the diaphragm is classified as T4 disease.^2^ Multiplanar reconstructions (MPRs) using sagittal and coronal views should always be used to assess an infiltration of these structures, as axial reconstruction might understate diaphragmatic infiltration.^6^

Unequivocal signs of an infiltration include the evidence of tumour masses that have infiltrated neighbouring structures. In case of the chest wall, the best evidence are rib erosions or a broad extension of tumour masses into the intercostal space.

Less reliable signs of an infiltration of neighbouring structures are impaired respiratory movement or a thickening of the pleura. Both of these signs, however, have only a limited positive predictive value, as inflammatory reactions around tumours may also result in the same signs.

MRI, especially using dynamic CINE sequences, has a higher accuracy (77%) compared to CT (47%) for the evaluation of chest wall and diaphragm infiltration.^7^ In particular, the sensitivity of up to 100% for MRI is superior compared to the 60% sensitivity of conventional CT. Tumours that infiltrate the diaphragm and/or chest wall do not follow the normal respiratory movement.^7^ This can be visualised on MRI and used for staging purposes. Similarly, dynamic CT examinations have higher sensitivity and specificity compared to conventional static CT.^8^

Superior sulcus tumours (Pancoast tumours) are classified as at least T3 tumours because of their chest wall infiltration. In the presence of invasion of the brachial plexus above C8, the vertebral body, spinal canal, or subclavian vessels, these tumours are upstaged to T4.

### Infiltration of the mediastinum

An infiltration of the mediastinum is defined as T4. The mediastinal structures that define T4 are the mediastinal fat, great vessels, the oesophagus, the trachea, and the heart. While an unequivocal invasion of the mediastinal fat is defined as T4, an invasion of the mediastinal parietal pleura alone is defined as T3.^2^ Therefore, tumour contact with the mediastinal pleura without direct or indirect signs of invasion is not automatically staged as T4. Tumour contact with a length of more than 3 cm or an obtuse angle between the tumour and the mediastinum are indirect signs of mediastinal infiltration. In contrast, direct infiltration of the mediastinal fat or of the structure contained within the mediastinum (*e.g.* heart, oesophagus) are staged as T4.^2^ The sensitivity and specificity for the assessment of mediastinal invasion by CT ranges from 40 to 78% and 69 to 99% (summarised in Seo et al,^9^ respectively), while it is up to 100 and 93% for cine MR images.^9^

**Figure 4.**
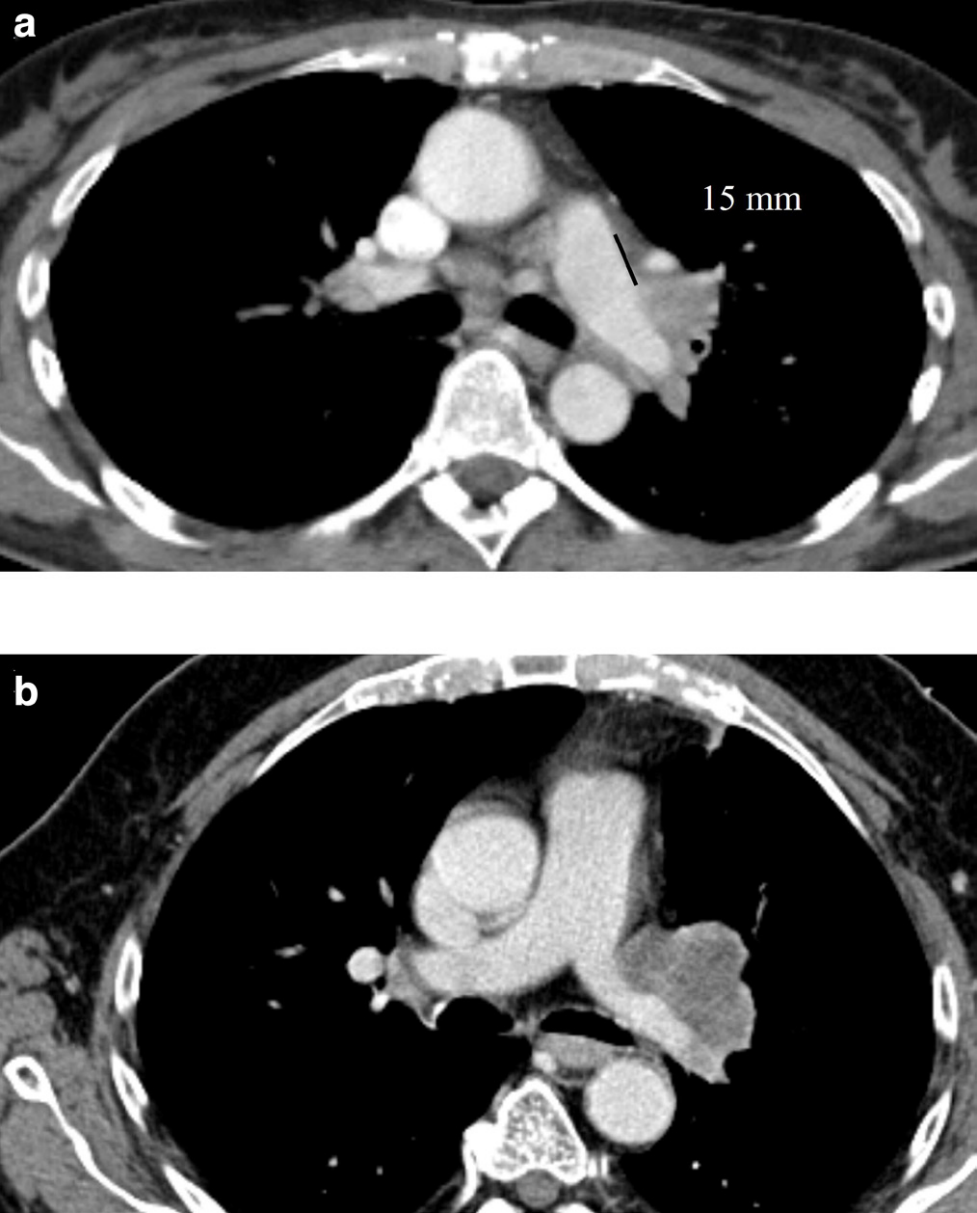
(a) Axial contrast-enhanced CT of a 55-year-old female patient with an NSCLC at the left hilum. As the distance between the main pulmonary trunk and the tumour is more than 10 mm, it is classified as T3 disease. (b) In contrast, in this 73-year-old male patient with NSCLC, the tumour is in direct contact with the main pulmonary artery and left main pulmonary artery, and therefore, staged as T4. NSCLC, non-small cell lung cancer.

In the staging system, great vessels are defined as the aorta, the superior and inferior vena cava, the main pulmonary trunk, and the intrapericardial portions of the pulmonary arteries and veins. While the aorta, the superior and inferior vena cava, as well as the main pulmonary trunk can be identified easily on CT, the border between the intra- and extrapericardial portions of the pulmonary arteries and veins cannot, because the pericardial fold cannot be visualised with CT. The pericardial fold around the right pulmonary artery may be defined by an imaginary line at the mid-half of the superior vena cava, while the pericardial fold of the left pulmonary artery is roughly 1 cm from the bifurcation of the main pulmonary artery (Figure 4).

### Intrapulmonary metastases

Intrapulmonary metastases are classified based on their location. Pulmonary metastases in the same lobe as the primary tumour are classified as T3, while metastases in another lobe on the same side as the primary as T4, and metastases to the contralateral lung as M1a.^2^

While this classification of pulmonary metastases sounds straightforward, it may lead to overstaging if the suspected pulmonary metastases are not verified histologically, as most pulmonary nodules detected in patients with lung cancer are benign.^10^ Consequently, additional pulmonary nodules that might have an impact on treatment should be verified histologically to determine whether they are malignant.

If malignant, the radiologist has to discriminate intrapulmonary metastases from a second primary.

**Figure 5.**
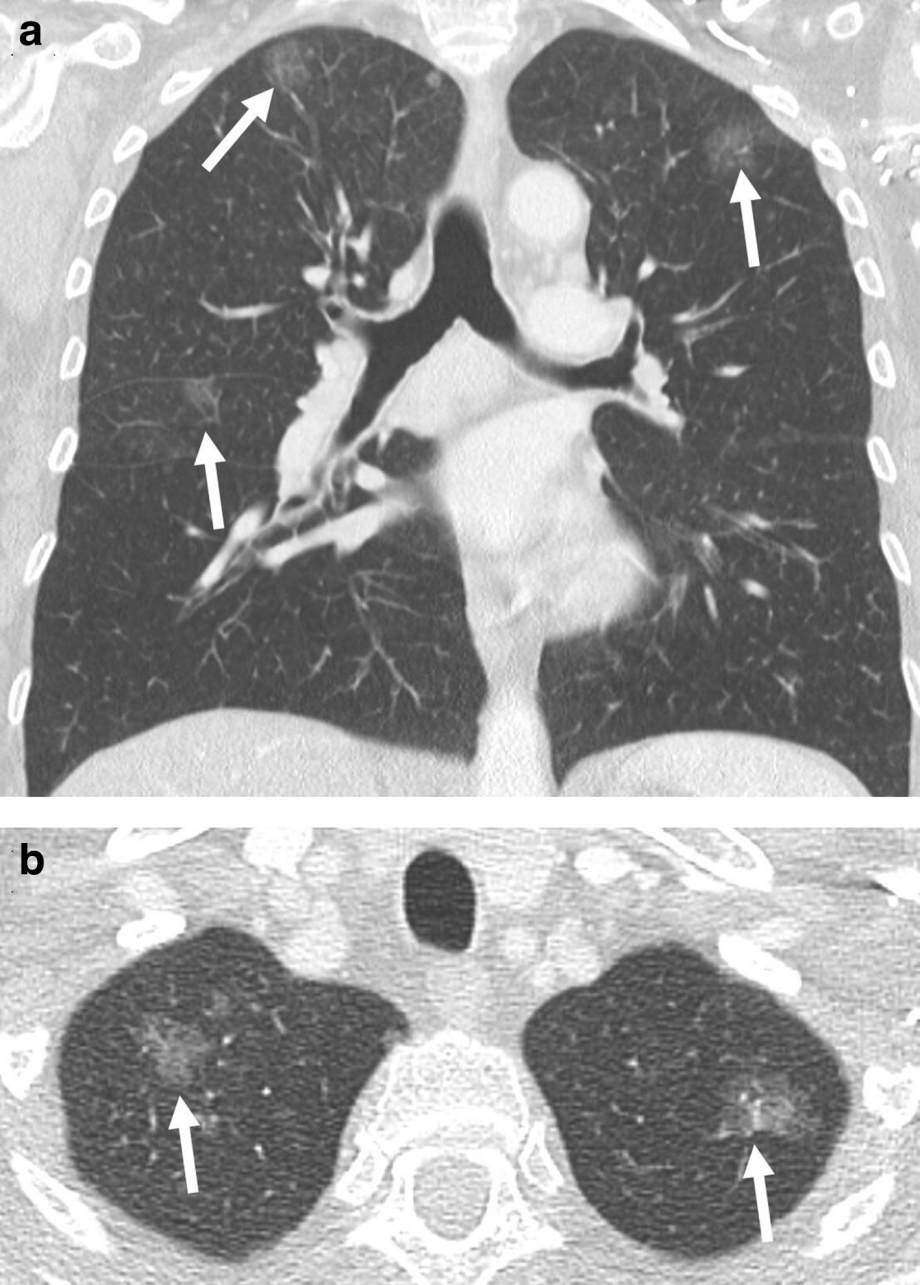
(a) Coronal and (b) axial contrast-enhanced CT of a female patient with three pure ground-glass nodules that represent *in situ* adenocarcinomas in the right and left upper lobe, as well as the middle lobe (arrow). The correct staging is based on the tumour dimension of the largest ground-glass nodules (3.3 cm), as well as the number of tumours, which is given in brackets: T2a(3)N0M0.

Table 1 summarises the radiological criteria by which to distinguish a second primary from a metastasis.^10^ Intrapulmonary metastases should be considered for solid lung cancers that have a separate tumour nodule(s) with a similar solid appearance and with (likely) matching histologic appearance (Figure 5).

**Table 1.** Criteria by which to distinguish second primary tumours *vs* metastasis (adapted from Detterbeck t al^10^)

Relative criteria that favour synchronous tumours
Different radiography appearance (*i.e.* shape, density)
Different metabolic activity
Different growth rates (if previous imaging is available)
Absence of nodal or systemic metastasis
Relative criteria that favour metastasis
Same radiographic appearance
Similar growth rates (if previous imaging is available)
Significant nodal or systemic metastases

Second primary lung cancers should be staged separately, each with a T, N, and M descriptor. For example, a patient with a 3.5 cm adenocarcinoma of the left upper lobe, a 2.4 cm squamous cell carcinoma of the right lower lobe, and with a metastatic lymph node in R11 without evidence of systemic metastasis, should be classified as having a T2aN0M0 adenocarcinoma and a T1cN1Mo squamous cell carcinoma.

It is important to note that the above-mentioned guidance for the staging of primary lung cancer should not be applied in patients with multiple subsolid tumour nodules (either pure GG nodules or part-solid nodules) or in patients with pneumonic-type lung cancer. For patients with multifocal GG or part-solid nodules, the T descriptor is based on the highest T lesion (#/m) indicating the number of tumour nodules. The T(#/m) classification should be applied independently whether the GG/part-solid lesions are in the same or in different ipsi- or contralateral lobes. In contrast, for pneumonic-type lung adenocarcinoma, the T is based on size for T3 if the tumour is limited to one single lobe, and T4 or M1a if in different ipsilateral or contralateral lobes, respectively. Both tumour types use a single N and M staging, regardless of how many GG/part-solid nodules are present.

Table 2 summarises potential pitfalls that can occur during the T-descriptor assessment and solutions to overcome these limitations.

**Table 2.** Pitfalls for assessing the T stage

Pitfall	Effect	Solution
Thick slices for T-descriptor assessment (1.5–5 mm)	The solid component in a part-solid tumour could be missed → misclassification as pure ground-glass tumours	Thin slices (1 mm)
Axial reconstructions only	Potentially not capturing the single largest tumour dimension→ low T descriptor	MPR in axial, sagittal, and coronal reconstructions to assess single largest tumour dimension
Using non-lung window settings without sharp filter	Underestimation of tumour dimension → low T descriptor	Lung-window setting with a sharp filter
Assessing diaphragmatic infiltration on axial reconstructions	Diaphragmatic infiltration might be missed → low T descriptor	Sagittal and coronal reconstructions for assessment of diaphragmatic infiltration

MPR, multiplanar reconstruction.

### Nodal disease

The N-descriptor remained unchanged in the eighth TNM edition compared to the seventh edition. Lymph node metastases to the ipsilateral hilum are classified as N1, lymph node metastases to the ipsilateral mediastinum or the subcarinal lymph nodes as N2, and contralateral mediastinal or supraclavicular lymph node metastases as N3.

Although CT is routinely used for the initial staging of patients with lung cancer, it has limited accuracy for the detection of thoracic lymph node metastases, as a short axis equal to or larger than 1 cm of the lymph nodes is the only criterion used to diagnose lymph node involvement. However, as also benign lymph nodes, such as inflammatory lymph nodes, may exceed this threshold, this criterion has only a low diagnostic value. In a metanalysis, it could be shown that the pooled sensitivity of the size criterion is 55%, with a sensitivity of 81%.^11^ By using this criterion only, 42% of lymph nodes larger than 1 cm would be overstaged, as they are benign, and 17% of metastases in lymph nodes smaller than 1 cm would be missed.^12^

By using fludeoxyglucose positron emission tomography (FDG PET)/CT, the sensitivity could be increased to 80%, with a specificity of 88%.^11,13^ Consequently, PET/CT is recommended for patients planning to undergo curative therapy (surgery or radiotherapy) to exclude lymph node involvement.^14^ However, as FDG PET/CT also struggles with false-positive results, histological confirmation of enlarged and/or FDG-positive lymph nodes is required to confirm metastatic involvement.^15^ A negative FDG PET in normal-sized hilar or mediastinal lymph nodes in patients with a small tumours (<3 cm) virtually excludes lymph node metastases, and thus, further invasive staging is not necessary. However, in larger (≥3 cm) central tumours, even a negative FDG PET/CT does not exclude lymph node metastases and invasive staging should be performed.^15–17^

Equally important to the diagnosis of lymph node involvement is the correct assignment of the lymph node location. Based on the anatomic location, the involved lymph nodes are assigned to lymph node stations that are defined in the TNM atlas.^18^ Common pitfalls in the assignment of the appropriate lymph node station are the assignment of right *vs* left paratracheal lymph nodes.^19^ Importantly, the border between the right and left paratracheal lymph nodes is not the midline of the trachea, but the left lateral wall of the trachea.^18^

**Figure 6.**
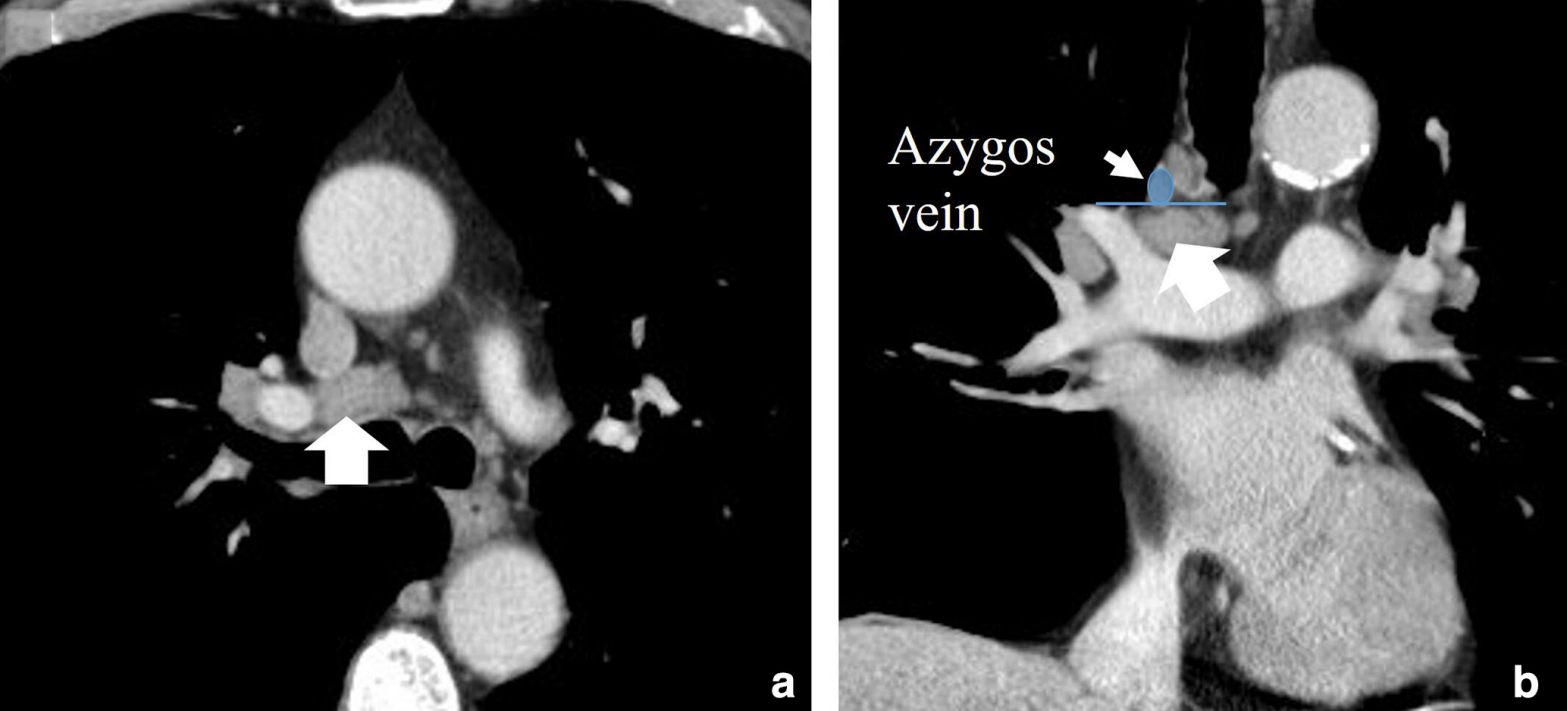
(a) Axial and (b) coronal contrast enhanced CT showing and enlarged (>10 mm) lymph node in the station 10 on the right sight (10R). Please note that the lymph node is below the level of the azygos vein.

A common misclassification of lymph nodes can also occur at the border between the lower paratracheal lymph nodes and hilar lymph nodes. On the right side, the lower aspect of the azygos vein separates the lower paratracheal lymph nodes from the hilar lymph nodes (Figure 6); on the left side, this border is defined by the upper aspect of the main pulmonary artery.^18^ In case of doubt, coronal reformations are helpful to identify the anatomic landmarks to determine the appropriate lymph node station.

**Figure 7.**
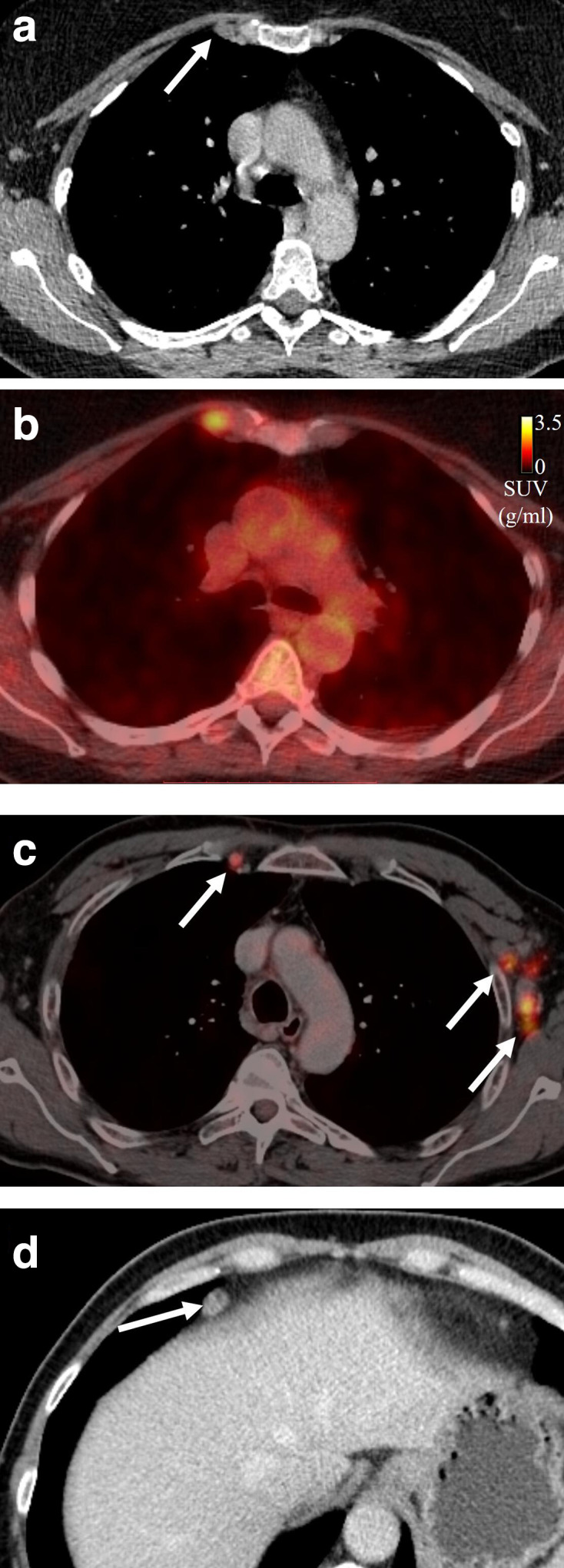
Lymph node stations that are not specified in the eighth TNM manifest include (a, b, c) internal mammary nodes, (c) axillary nodes, and (d) diaphragmatic nodes (arrows). They can either be classified as N3 or M1 disease.

Some lymph node stations in the thorax (*i.e.* anterior, middle, and posterior diaphragmatic nodes, intercostal nodes, internal mammary nodes, retrocrural nodes, and axillary nodes) are not included in the International Association for the Study of Lung Cancer lymph node map at all. Therefore, it is unclear whether they are classified as N3 or M1 disease (Figure 7). Axillary lymph node metastases are seen in <1% of patients with NSCLC at the time of presentation and are associated with other M1 features in ~50% of cases.^20^ In the authors’ personal experience, however, lymph node metastasis in the above-mentioned lymph node stations rarely occurs without other evidence of advanced tumour stage (*i.e.* tumour size, lymph node metastasis, distant metastasis) that drive the tumour staging.

### Metastatic disease

Approximately 20–50% of patients with lung cancer have distant metastases at the time of initial staging, with bone, brain, adrenal, and liver being the most common locations for metastasis.^21^ Importantly, some studies have shown that metastasis can occur at the time of angiogenesis when lesions are as small as 1–2 mm.^22,23^

In the TNM staging system, distant metastases are described using the M descriptor.

The M1a category describes intrathoracic metastases, such as one or more additional nodules in the contralateral lung or a tumour with malignant pleural or pericardial nodules or effusion.

### Malignant pleural or pericardial effusion

Malignant pleural or pericardial effusion and or pleural or pericardial metastases are defined as M1a. Up to 16% of patients with NSCLC have malignant pleural effusion at presentation.^24^ The sensitivity and specificity of CT for reporting malignant pleural effusion are 62–75%, and 72–78%, respectively.^25^ Imaging features that suggest malignancy are nodular pleural thickening, mediastinal pleural thickening, and parietal pleural thickening >1 cm. However, with a negative predictive value of ~65%, approximately one in every three patients with suspected malignant pleural disease and pleural effusion, without any other radiological signs of malignancy, will have underlying malignant disease.^25^ Thus, in case of equivocal findings on CT, patients should undergo pleural tap or invasive pleural biopsies.

### Extrathoracic metastases

Extrathoracic metastases are subdivided into two subcategories, namely, M1b and M1c. The M1b category describes one solitary extrathoracic metastases in one single organ. M1c is defined by the presence of two or more extrathoracic metastases. The subclassification of extrathoracic metastases into M1b and M1c was introduced in the eighth edition of the TNM staging system, acknowledging the difference in survival rates between M1b and M1c.^26^

The reclassified descriptors not only provide an enhanced definition of metastatic disease and have a better ability to predict prognosis, but also maintain the compatibility with the previous existing descriptors of the seventh edition.

PET-CT proved very informative about metastatic spread in NSCLC, with an ability to detect unsuspected metastasis in up to 28% of patients, and also had an impact on management in up to 53% of patients.^27^

### Bone metastases

Approximately 35% of patients with lung cancer will develop bone metastasis during the course of their disease.^28^

CT delivers a high spatial resolution of cortical and trabecular bone to detect bone metastases. The availability of dedicated bone algorithms in acquisition protocols, the ability to adjust the window width and level, and the possibility of MPRs results in a higher sensitivity for CT compared to plain radiography in detecting both osteolytic and osteosclerotic metastases. Bone metastasis in lung cancer can be osteolytic, osteoblastic, or mixed, but are predominantly lytic on CT imaging. All morphologic types of baseline metastatic lesions may become sclerotic lesions if there is a therapeutic response. In contrast, if a therapeutic response is not completely achieved, different patterns are recognised. A meta-analysis showed a higher sensitivity (92% *vs* 87%) and specificity (98% *vs* 94%) for the detection of bone metastases when CT was combined with ^18^F-FDG PET.^29^

**Figure 8.**
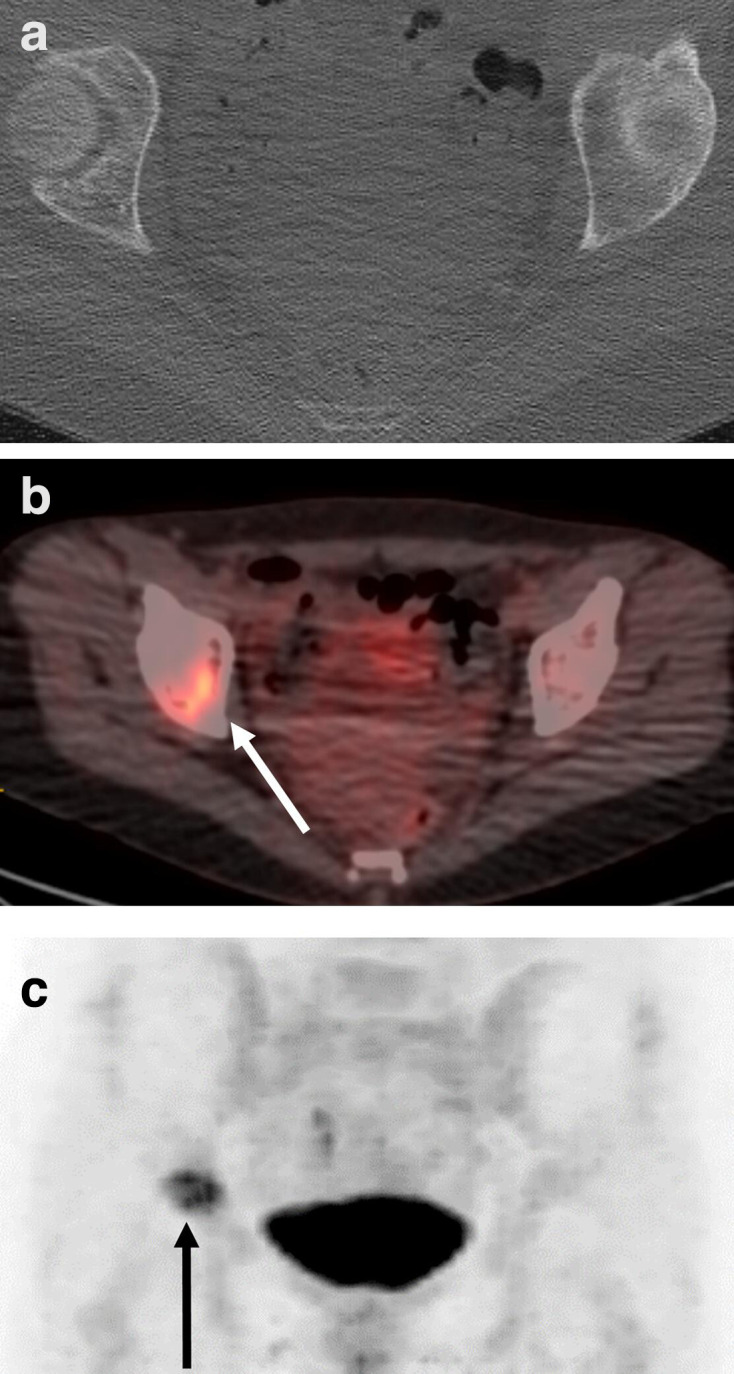
Axial contrast-enhanced FDG-PET/CT of a 59-year-old female patient with NSCLC. The bone metastasis in the right acetabulum was not visible on the (a) CT, while it was clearly visible on the (b, c) FDG-PET (arrow). The accuracy of the FDG-PET/CT to detect bone metastasis is higher compared to CT alone. FDG, fludeoxyglucose;PET, positron emission tomography.

The accuracy of bone scintigraphy in the detection of bone metastasis was 87 *vs* 98% for FDG-PET/CT. PET scanning is more sensitive and accurate than bone scanning for the detection of skeletal metastases (Figure 8), and is, therefore, recommended for all patients scheduled for curative surgery to exclude distant metastases.^14,30^

Bone islands can mimic sclerotic metastasis on CT imaging, but can usually be differentiated by their high attenuation values measured on CT. Another commonly encountered pitfall are haemangiomas of the vertebral body. The classical polka-dot appearance of haemangiomas may not be present ubiquitously. Some haemangiomas may exhibit a tracer uptake on PET-CT and this may further add to confounding clinical questions. In a majority of these instances when PET findings are positive and CT is negative, MR imaging may be the problem-solving tool. Both T1 and more advanced techniques as a modified Dixon TSE-T2 sequences show excellent sensitivity and specificity for the detection of bone metastasis.^31^ False-negative results on FDG PET/CT may be seen in the case of osteoblastic formations, as bone matrix proliferation reduces the glycolytic activity essential for FDG uptake.

### Adrenal gland metastases

Metastasis to the adrenal glands is common in patients with NSCLC and is usually accompanied by metastases in other organs, although it can present as oligometastatic disease.^32^ It is essential to distinguish commonly occurring benign entities of the adrenal gland, such as adenomas, from a metastatic lesion. The prevalence of adrenal adenoma is reported to be related to age; the frequency of unsuspected adenoma is 0.5% in patients aged 20–29 years and 7% in those older than 70 years.^33^

In lipid-rich adenomas, the non-invasive diagnosis relies on the proof of fat components. On CT, attenuation values below 10 Hounsfiedl unit (HU) in regions of interest (ROIs) encompassing two-thirds of the circumference of the region are highly specific for adenomas.^33^

As one-third of adrenal adenomas have a low lipid content, CT attenuation values above 10 HU do not exclude adenomas. To diagnose lipid-poor adenomas, contrast-enhanced CT scans or MRI are performed. MRI using chemical shift imaging has a higher sensitivity for the detection of lipids than CT, and is thus indicated in adrenal lesions with attenuation values between 10 and 20 HU.^33^ Combined chemical-shift and dynamic-MR imaging was shown to have a sensitivity of 91% and a specificity of 94% in the differentiation between benign and malignant adrenal lesions.^34^ For adenomas with CT attenuation values greater than 20 HU, washout CT is superior to chemical shift imaging.^33^

**Figure 9.**
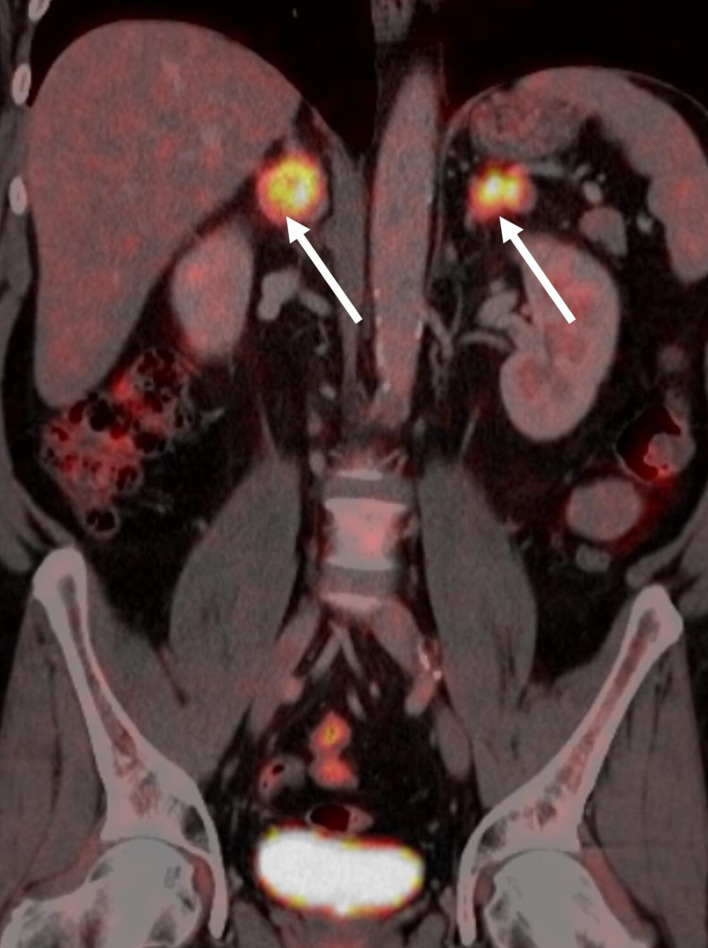
Coronal FDG PET/CT scan showing metabolically active, bilateral adrenal gland metastasis (arrow). FDG, fludeoxyglucose;PET, positron emission tomography.

**Figure 10.**
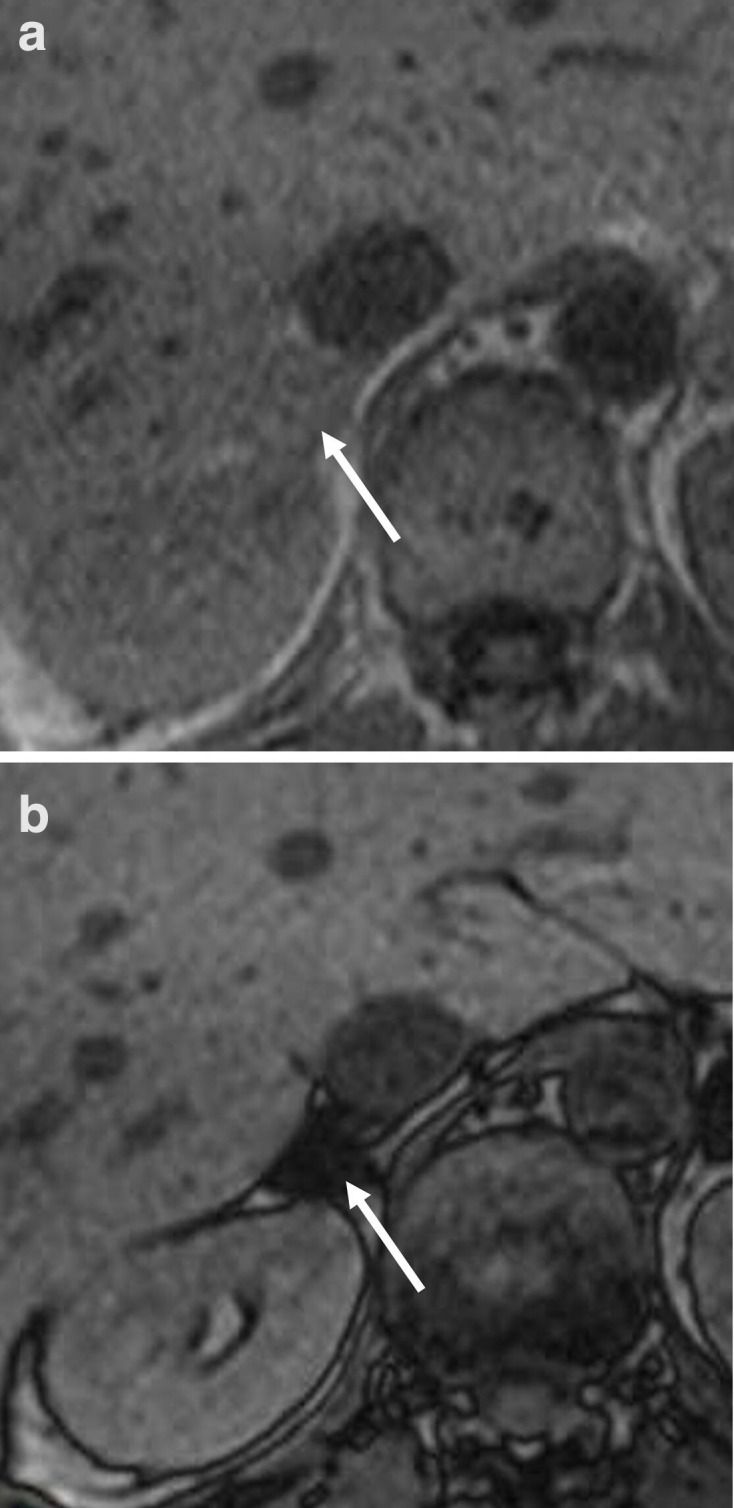
Axial a) in and b) opposed phase MRI images showing a right sided adrenal adenoma (signal drop in the opposed phase comared to the in phase.

PET/CT and MRI are useful in distinguishing benign and malignant adrenal masses (Figures 9 and 10). In a metanalysis of nine studies evaluating the accuracy of FDG PET/CT for the detection of adrenal metastases in patients with NSCLC, the sensitivity was 89%, the specificity was 90%, the positive likelihood ratio was 8.5, and the negative likelihood ratio was 0.09.^35^ False-negative FDG PET/CT findings may be the result of haemorrhage or necrosis in a metastasis measuring less than 1.0 cm.^36^

### Brain metastases

**Figure 11.**
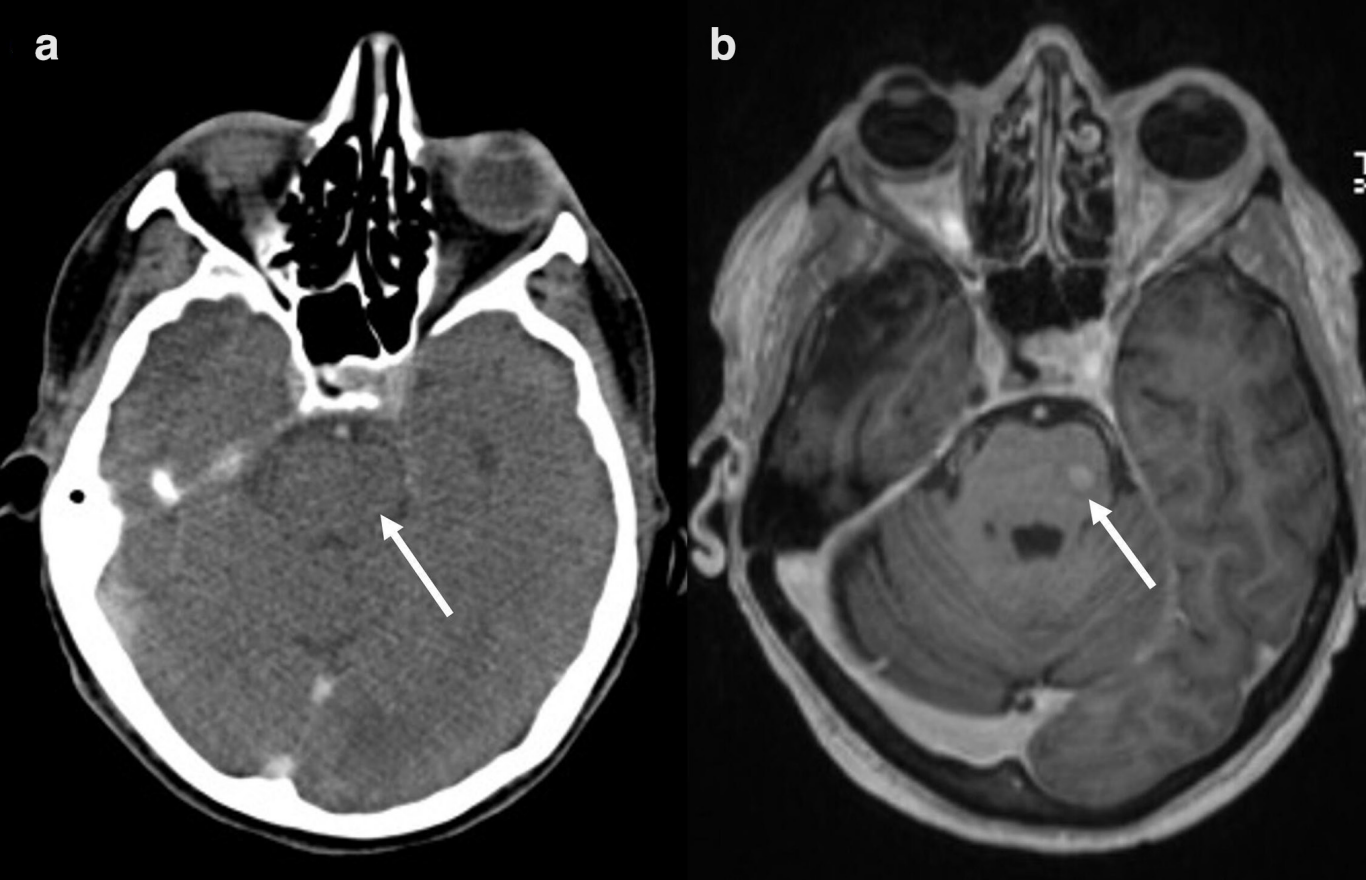
Axial a) contrast-enhanced CT and b) MRI of the neurocranium in a patient with newly diagnosed adenocarcinoma who was neurologically asymptomatic at the time of imaging. The CT scan showed no abnormalities, whereas there was a focal, increased contrast uptake in the left brainstem highly suggestive of a cerebral metastasis. The lesion was proven to be malignant in the follow-up examination by the pattern of growth.

Contrasted-enhanced MRI has a higher sensitivity for the detection of brain metastasis compared to CT and PET. In the March 2019 revisions of the NICE guideline, contrast-enhanced CT in patients with Stage II NSCLC having treatment with curative intent is recommended.^37^ In contrast, in patients with Stage III NSCLC having treatment with curative intent contrast-enhanced MRI is recommended. In addition, in patients with neurological symptoms, MRI of the brain should be performed (Figure 11) .^38^

### Liver metastases

Isolated liver metastases have been reported in approximately 3–4% of NSCLC patients.^39^ Although the characteristic CT appearance in a triple-phase scan is a hypodense, hypoenhancing lesion, a subset of liver metastases may rarely manifest as hyperenhancing lesions, particularly when there is small cell neuroendocrine differentiation in the primary lung cancer. This may sometimes be confounded with a commonly occurring benign entity of the liver, such as a haemangioma. Although MRI has a higher accuracy in the detection of liver metastases compared to CT,^40^ it is not routinely recommended.^38^

### Soft tissue metastases

The lung, followed by the kidney and the colon, is the most common primary carcinoma site that leads to clinically recognised soft-tissue metastases.^41^ Relevant differentials of soft tissue metastases include injection site granulomas, atheromas, and neurogenic tumours. On PET-CT, false-positive results may be caused by foci of brown fat, but the typical location at the neck, paravertebral, mediastinal, and axillary regions may provide a clue to the diagnosis.

## Conclusion

The TNM in its eighth edition provides a detailed framework by which to describe the anatomical extent of disease in patients with lung cancer. Despite several advantages compared to the former version, some limitations remain. It is important for radiologists to recognise these limitations.^42^ In those cases, radiologists should be aware of the following general rule stated in the manuscript. “If there is doubt concerning the correct T, N, or M category to which a particular case should be allotted, the lower (*i.e.* less advanced) category should be chosen”.^43^ Applying this rule in case of uncertainty could give patient the choice of doubt and enable the attempt of a curative treatment.
